# Crystal structure of di­chlorido­bis­{μ-2-meth­oxy-6-[(methyl­imino)­meth­yl]phenolato}{2-meth­oxy-6-[(methyl­imino)­meth­yl]phenolato}cadmium(II)cobalt(III) monohydrate

**DOI:** 10.1107/S2056989018013610

**Published:** 2018-10-02

**Authors:** Olga Yu. Vassilyeva, Katerina V. Kasyanova, Vladimir N. Kokozay, Brian W. Skelton

**Affiliations:** a Department of Chemistry, Taras Shevchenko National University of Kyiv, 64/13 Volodymyrska Street, Kyiv 01601, Ukraine; b School of Molecular Sciences, M310, University of Western Australia, Perth, WA 6009, Australia

**Keywords:** crystal structure, heterometallic complex, Schiff base ligand, *o*-vanillin, methyl­amine

## Abstract

The title compound, [CoCd(C_9_H_10_NO_2_)_3_Cl_2_]·H_2_O, is a solvatomorph of the corresponding hemihydrate recently published by us [Nesterova *et al.* (2018 ▸). *Appl. Cat. A*, **560**, 171–184]. The current structure reveals different cell parameters and space group compared with the published one while both are monoclinic with almost the same cell volume. The title compound is formed of discrete neutral dinuclear mol­ecules with no crystallographically imposed symmetry and water mol­ecules of crystallization. The overall geometry about the cobalt(III) ion is octa­hedral with an N_3_O_3_ environment; each ligand acts as a meridional ONO donor. The Cd^II^ coordination sphere approximates an irregular square pyramid with a chlorine atom at the apex. There is significant shortening of a Cd—O bond length to the oxygen atom of the methoxo group on one of the ligands [2.459 (3) Å] compared to the corresponding distance in the published structure [2.724 (7) Å], while other Cd—Cl/N/O bonds remain roughly the same. In the crystal lattice, the heterometallic mol­ecules, which are related by the crystallographic *n*-glide plane and inter­linked by weak hydrogen bonds to solvent water mol­ecules, form columns along [101]. Adjacent columns lie anti­parallel to each other.

## Abstract 2

Heterometallic Co/Cd solvatomorphs, which differ by half of the solvent water mol­ecule, show a remarkable variation in the cadmium coordination sphere.

## Chemical context   

The title compound, [CoCd(C_9_H_10_NO_2_)_3_Cl_2_]·H_2_O, (**1**) is a solvatomorph of the corresponding hemihydrate recently published by us (CSD refcode TEZKER; Nesterova *et al.*, 2018 ▸). We have studied the heterometallic hemihydrate [CoCd*L*_3_Cl_2_]·0.5H_2_O (**2**) with a Schiff base ligand {H*L* is 2-meth­oxy-6-[(methyl­imino)­meth­yl]phenol} and its related complex [Co*L*_3_]·DMF (DMF is *N*,*N*′-di­methyl­formamide) in alkanes oxidation reactions. Complexes of transition metals have proved to be efficient catalysts for a broad range of organic reactions, including direct C—H functionalization (Pototschnig *et al.*, 2017 ▸; Nesterov *et al.*, 2018 ▸). At the same time, the catalytic properties of heterometallic compounds, and those combining catalytically active and non-active metals in particular, in stereospecific *sp* ^3^ C—H oxidation with *m*-chloro­perbenzoic acid have received significantly less attention. A comparison of the catalytic behaviours of the hetero- and monometallic analogues provided further insight into the origin of stereoselectivity of the oxidation of C—H bonds (Nesterova *et al.*, 2018 ▸).
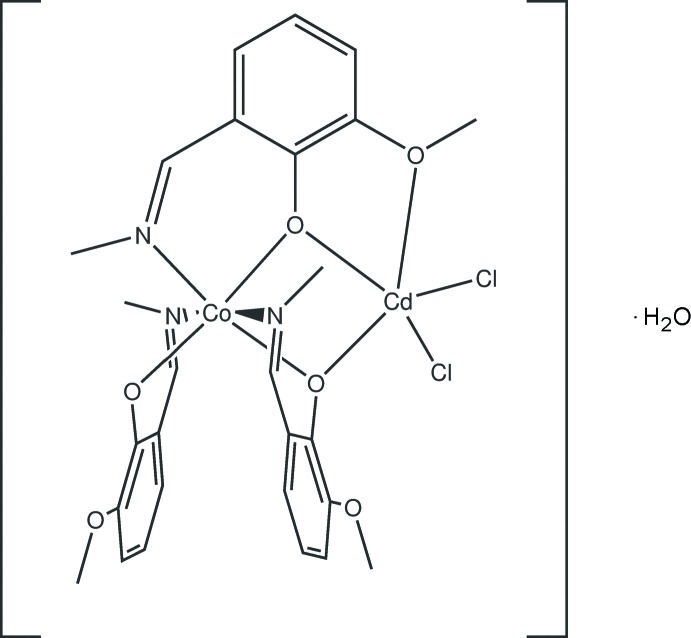


While the hemihydrate (**2**) was prepared by *direct synthesis* (Kokozay *et al.*, 2018 ▸) employing Co powder and cadmium chloride as starting materials, for the synthesis of the title compound two metal acetate salts were reacted with the Schiff base formed *in situ* from the condensation between *o*-vanillin and CH_3_NH_2_·HCl in water/ethanol in a 1:1:3 molar ratio. Remarkably, the isolated plate-like crystals of (**1**) were brown–red, not brown–green, and appeared non-isostructural with the prismatic hemihydrate (**2**) while both are monoclinic with almost the same cell volume [2923.10 (10) Å^3^ in (**1**) and 2931.3 (7) Å^3^ in (**2**)]. The previously published structure was solved and refined in the standard setting *P*2_1_/*c* whereas the current structure is in *P*2_1_/*n* [*a*, *b*, *c*, *β*: 9.4036 (2), 21.1588 (4), 15.0319 (3) Å, 102.221 (2)°, respectively in (**1**) and 14.090 (2), 16.887 (2), 13.179 (2) Å, 110.84 (2)° in (**2**)]. Another striking difference is a significantly shorter Cd—O bond length to the oxygen atom of the methoxo group on one of the ligands [2.459 (3) Å] compared to the corresponding distance in (**2**) [2.724 (7) Å], while other Cd—Cl/N/O bonds remain roughly the same. The reason for such a discrepancy could be the incorporation of a whole water mol­ecule in (**1**) instead of a half-mol­ecule in (**2**), which slightly changes the hydrogen bonding and packing motifs in the former compound.

## Structural commentary   

**Figure 1:**
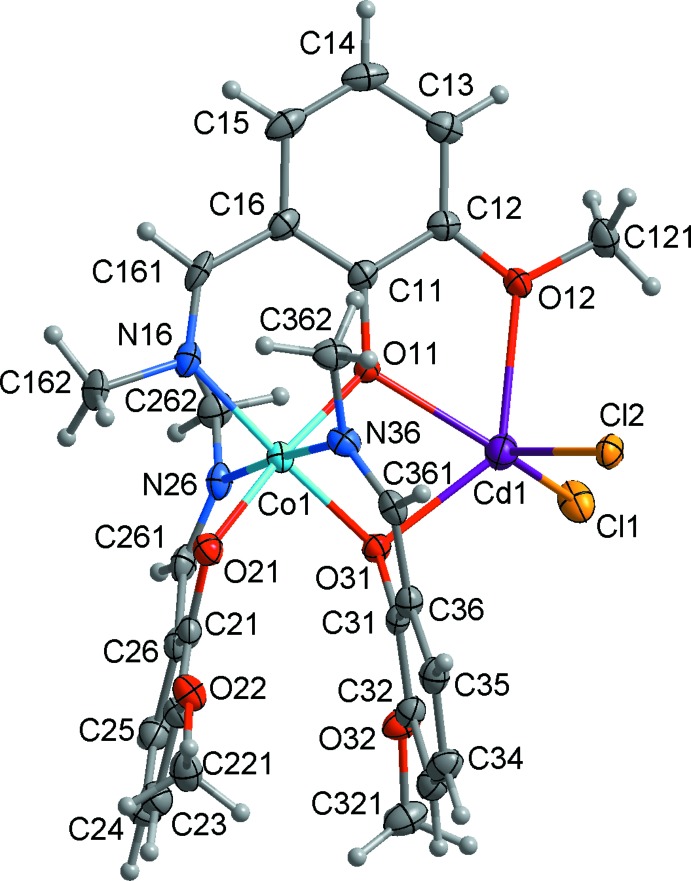
The mol­ecular structure of the title compound, showing the atom-numbering scheme. Non-H atoms are shown with displacement ellipsoids at the 30% probability level.

The heterometallic complex (**1**) is built up from discrete CoCd*L*_3_Cl_2_ mol­ecules and water mol­ecules of crystallization. The mol­ecular structure of (**1**) closely resembles that of the hemihydrate (Nesterova *et al.*, 2018 ▸). The complex mol­ecule has no crystallographically imposed symmetry (Fig. 1 ▸). The ligand moieties are deprotonated at the phenol O atom and octa­hedrally coordinate the Co^III^ ion through the three azomethine N and three phenolate O atoms in a *mer* configur­ation. The three crystallographically non-equivalent salicyl­aldimine ligands have Co—O and Co—N bond lengths in the ranges 1.871 (4)–1.932 (3) and 1.933 (4)–1.961 (5) Å, respectively, (Table 1 ▸). Average Co—O and Co—N bond lengths in (**1**) and (**2**) are almost equal, being 1.905 and 1.945 Å, respectively, in the monohydrate and 1.900 and 1.945 Å in the hemihydrate. The *trans* angles at the cobalt atom vary from 173.23 (16) to 176.3 (2)° while the *cis* angles are in the range 82.67 (14)–93.70 (19)° (Table 1 ▸).

The nearest coordination geometry of the cadmium centre in (**1**) is strictly comparable to that for (**2**). The cadmium atom has two quite short bonds with the bridging phenolato oxygen atoms, O11 and O31 [2.235 (3), 2.286 (3) Å], of the two deprotonated Schiff bases and two longer bonding distances to the chlorine atoms [Cl1: 2.4091 (14), Cl2: 2.4222 (12) Å] in a distorted tetra­hedral geometry. The angles at the metal atom vary from 68.33 (12) to 127.90 (10)° (Table 1 ▸). In addition, Cd1 is weakly bonded to the oxygen atom O12 at 2.459 (3) Å, which implies that the Cd1 coordination sphere approximates an irregular square pyramid with Cl1 atom at the apex. There is a marked decrease in the Cd—O12 bond length when (**1**) is compared to (**2**) [2.724 (7) Å] and the cobalt–cadmium separation [3.286 Å in (**1**) *versus* 3.315 Å in (**2**)], providing a rare structural example of variations in the metal coordination sphere to accommodate changes possibly caused by a different number of solvent mol­ecules in the crystal lattice.

## Supra­molecular features   

**Figure 2:**
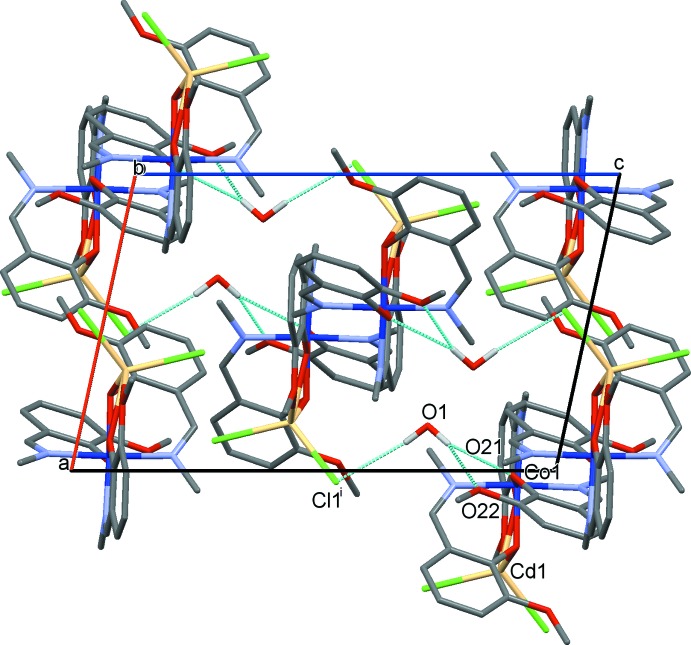
Crystal packing of (**1**) showing columns of CoCd*L*_3_Cl_2_ mol­ecules joined by hydrogen-bonding inter­actions through the solvent water mol­ecules along [101]. The symmetry code is as in Table 2 ▸. Hydrogen bonds are shown as blue dashed lines. Only water H atoms are shown.

The heterometallic mol­ecules related by the crystallographic *n*-glide plane are stacked along [101] with adjacent columns lying anti­parallel to each other (Fig. 2 ▸). The dinuclear units show no significant inter­molecular inter­actions in the solid state: the minimum Co⋯Cd separation between the neighbouring CoCd*L*_3_Cl_2_ mol­ecules within a column is 8.372 Å. There are weak hydrogen bonds between the solvent water mol­ecule and the oxygen atoms on one of the ligands (O21, O22) and also to the Cl1 atom of the mol­ecule related by the crystallographic *n*-glide plane (Fig. 2 ▸, Table 2 ▸). Very weak C—H⋯Cl/O hydrogen-bonding inter­actions between the complex mol­ecules lead to a consolidation of the crystal packing.

## Database survey   

A search of the Cambridge Structural Database (CSD; Groom *et al.*, 2016 ▸) *via* the WebCSD inter­face in September 2018 returned 43 hits for the crystal structures of metal complexes with H*L* and the ligand itself. Almost half of the complexes are hepta­nuclear homometallic assemblies (*M* = Mn, Co, Ni, Zn) with planar hexa­gonal disc-like cores and varying anions and solvent mol­ecules. The metal centres in the cores are in distorted octa­hedral geometries with the six μ_3_-bridging OH^−^ or MeO^−^ ions linking the central metal atom to the six peripheral ones; the metal-to-ligand ratio *M* ^II^:*L* is 7:6. The ligand mol­ecules are singly deprotonated at the phenolate site and adopt a chelating bridging mode, forming five- and six-membered rings similar to those in (**1**). The rest of the complexes are mainly mononuclear compounds with mol­ecular (Mn, Co and Pt) or polymeric (Mn) arrangements in the crystal lattice and metal-to-ligand ratios *M* ^II/III^:*L* of 1:2 and 1:3. There are also dimeric (Cu) and tetra­meric (Co, Mn) complexes with the teranuclear cores additionally supported by other bridging ligands. The heterometallic examples with H*L* are limited to the four Na/*M* (*M* = Fe, Ni) 1*s*–3*d* structures of 4 and 5 nuclearity and [CoCd*L*_3_Cl_2_]·0.5H_2_O (2) already mentioned.

## Synthesis and crystallization   

2-Hy­droxy-3-meth­oxy-benzaldehyde (0.23 g, 1.5 mmol) and methyl­amine hydro­chloride (0.10 g, 1.5 mmol) were added to ethanol (10 ml) and stirred magnetically for 10 min. Cd(CH_3_COO)_2_·2H_2_O (0.13 g, 0.5 mmol) and Co(CH_3_COO)_2_·4H_2_O (0.12 g, 0.5 mmol) both dissolved in 2 ml water were added to the light-yellow solution of the Schiff base formed *in situ.* The resultant red–brown solution was stirred at room temperature for an hour, then filtered and left to stand at room temperature. Brown–red plate-like crystals of (**1**) suitable for crystallographic characterization were formed over several days in a mixture with yellow flakes. They were collected by filter-suction, washed with ethanol and finally dried in air.

## Refinement   

Crystal data, data collection and structure refinement details are summarized in Table 3 ▸. The *P*2_1_/*n* setting is the obvious choice for (**1**) as this leads to a smaller β angle. The *P*2_1_/*c* setting of the current structure can be determined by the transformation [1 0 0, 0 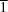 0, 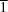 0 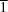] to give the unit cell *a* = 9.404, *b* = 21.159, *c* = 15.954 Å, *α* = *γ* = 90, *β* = 112.95°. It is clear that the unit cells of (**1**) and (**2**) are different even if both are compared in the standard *P*2_1_/*c* settings. The water mol­ecule hydrogen atoms in (**1**) were located and refined with geom­etries restrained to ideal values. All remaining hydrogen atoms were added at calculated positions and refined by use of a riding model with isotropic displacement parameters based on those of the parent atom (C—H = 0.95 Å, *U*_iso_(H) = 1.2*U*_eq_C for CH, C—H = 0.98 Å, *U*_iso_(H) = 1.5*U*_eq_C for CH_3_).

## Supplementary Material

Crystal structure: contains datablock(s) I, global. DOI: 10.1107/S2056989018013610/lh5882sup1.cif

Structure factors: contains datablock(s) I. DOI: 10.1107/S2056989018013610/lh5882Isup2.hkl

CCDC reference: 1869534

Additional supporting information:  crystallographic information; 3D view; checkCIF report

## Figures and Tables

**Table 1:** Selected geometric parameters (Å, °)

Cd1—O11	2.235 (3)	Co1—O11	1.913 (3)
Cd1—O31	2.286 (3)	Co1—O31	1.932 (3)
Cd1—Cl1	2.4091 (14)	Co1—N16	1.933 (4)
Cd1—Cl2	2.4222 (12)	Co1—N26	1.942 (4)
Cd1—O12	2.459 (3)	Co1—N36	1.961 (5)
Co1—O21	1.871 (4)		
			
O11—Cd1—O31	68.33 (12)	O21—Co1—N16	92.19 (18)
O11—Cd1—Cl1	127.90 (10)	O11—Co1—N16	92.91 (17)
O31—Cd1—Cl1	111.39 (9)	O31—Co1—N16	174.93 (18)
O11—Cd1—Cl2	116.80 (10)	O21—Co1—N26	92.72 (18)
O31—Cd1—Cl2	107.72 (9)	O11—Co1—N26	91.78 (17)
Cl1—Cd1—Cl2	112.53 (5)	O31—Co1—N26	88.21 (16)
O11—Cd1—O12	66.48 (12)	N16—Co1—N26	89.43 (19)
O31—Cd1—O12	134.52 (12)	O21—Co1—N36	85.17 (17)
Cl1—Cd1—O12	92.53 (9)	O11—Co1—N36	90.06 (17)
Cl2—Cd1—O12	96.82 (9)	O31—Co1—N36	88.82 (17)
O21—Co1—O11	173.23 (16)	N16—Co1—N36	93.70 (19)
O21—Co1—O31	92.41 (15)	N26—Co1—N36	176.3 (2)
O11—Co1—O31	82.67 (14)		

**Table 2:** Hydrogen-bond geometry (Å, °)

*D*—H⋯*A*	*D*—H	H⋯*A*	*D*⋯*A*	*D*—H⋯*A*
C361—H361⋯Cl1^i^	0.95	2.76	3.509 (5)	137
C362—H36*A*⋯O32^i^	0.98	2.45	3.204 (7)	134
O1—H1*AO*⋯O21	0.84 (6)	2.45 (7)	3.140 (6)	139 (9)
O1—H1*AO*⋯O22	0.84 (6)	2.19 (5)	2.965 (7)	153 (9)
O1—H1*BO*⋯Cl1^i^	0.84 (6)	2.54 (7)	3.368 (6)	173 (9)

Symmetry code: (i) 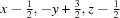.

**Table 3:** Experimental details

Crystal data	
Chemical formula	[CoCd(C_9_H_10_NO_2_)_3_Cl_2_]·H_2_O
*M*_r_	752.78
Crystal system, space group	Monoclinic, *P*2_1_/*n*
Temperature (K)	100
*a*, *b*, *c* (Å)	9.4036 (2), 21.1588 (4), 15.0319 (3)
β (°)	102.221 (2)
*V* (Å^3^)	2923.10 (10)
*Z*	4
Radiation type	Cu *K*α
μ (mm^−1^)	12.38
Crystal size (mm)	0.29 × 0.07 × 0.02

Data collection	
Diffractometer	Oxford Diffraction Gemini
Absorption correction	Analytical (*CrysAlis PRO*; Rigaku OD, 2015 ▸)
*T*_min_, *T*_max_	0.216, 0.808
No. of measured, independent and observed [*I* > 2σ(*I*)] reflections	25774, 5222, 4362
*R*_int_	0.060
(sin θ/λ)_max_ (Å^−1^)	0.599

Refinement	
*R*[*F* ^2^ > 2σ(*F* ^2^)], *wR*(*F* ^2^), *S*	0.046, 0.117, 1.02
No. of reflections	5222
No. of parameters	382
No. of restraints	2
H-atom treatment	H atoms treated by a mixture of independent and constrained refinement
Δρ_max_, Δρ_min_ (e Å^−3^)	2.10, −0.76

Computer programs: *CrysAlis PRO* (Rigaku OD, 2015 ▸), *SIR92* (Altomare *et al.*, 1994 ▸), *SHELXL2014* (Sheldrick, 2015 ▸), *DIAMOND* (Brandenburg, 1999 ▸). *Mercury* (Macrae *et al.*, 2006 ▸) and *WinGX* (Farrugia, 2012 ▸).

## supplementary crystallographic information

### Crystal data

**Table:**

[CoCd(C_9_H_10_NO_2_)_3_Cl_2_]·H_2_O	*F*(000) = 1520
*M**_r_* = 752.78	*D*_x_ = 1.711 Mg m^−^^3^
Monoclinic, *P*2_1_/*n*	Cu *K*α radiation, λ = 1.54178 Å
Hall symbol: -P 2yn	Cell parameters from 7300 reflections
*a* = 9.4036 (2) Å	θ = 3.7–66.9°
*b* = 21.1588 (4) Å	µ = 12.38 mm^−^^1^
*c* = 15.0319 (3) Å	*T* = 100 K
β = 102.221 (2)°	Plate, dark red
*V* = 2923.10 (10) Å^3^	0.29 × 0.07 × 0.02 mm
*Z* = 4	

### Data collection

**Table:**

Oxford Diffraction Gemini diffractometer	5222 independent reflections
Radiation source: sealed X-ray tube	4362 reflections with *I* > 2σ(*I*)
Mirror monochromator	*R*_int_ = 0.060
Detector resolution: 10.4738 pixels mm^-1^	θ_max_ = 67.3°, θ_min_ = 3.7°
ω scans	*h* = −7→11
Absorption correction: analytical (CrysAlis Pro; Rigaku OD, 2015)	*k* = −25→25
*T*_min_ = 0.216, *T*_max_ = 0.808	*l* = −17→17
25774 measured reflections	

### Refinement

**Table:**

Refinement on *F*^2^	2 restraints
Least-squares matrix: full	Hydrogen site location: mixed
*R*[*F*^2^ > 2σ(*F*^2^)] = 0.046	H atoms treated by a mixture of independent and constrained refinement
*wR*(*F*^2^) = 0.117	*w* = 1/[σ^2^(*F*_o_^2^) + (0.0562*P*)^2^ + 8.2206*P*] where *P* = (*F*_o_^2^ + 2*F*_c_^2^)/3
*S* = 1.02	(Δ/σ)_max_ = 0.001
5222 reflections	Δρ_max_ = 2.10 e Å^−^^3^
382 parameters	Δρ_min_ = −0.76 e Å^−^^3^

### Special details

**Table:**

Geometry. All esds (except the esd in the dihedral angle between two l.s. planes) are estimated using the full covariance matrix. The cell esds are taken into account individually in the estimation of esds in distances, angles and torsion angles; correlations between esds in cell parameters are only used when they are defined by crystal symmetry. An approximate (isotropic) treatment of cell esds is used for estimating esds involving l.s. planes.
Refinement. The water molecule hydrogen atoms were located and refined with geometries restrained to ideal values. The largest peak is 0.80 Angstroms from Cd1; the deepest hole is 0.73 Angstroms from Cd1.

### Fractional atomic coordinates and isotropic or equivalent isotropic displacement parameters (Å^2^)

**Table:**

	*x*	*y*	*z*	*U*_iso_*/*U*_eq_	
Cd1	0.83717 (3)	0.66924 (2)	0.43122 (2)	0.02764 (12)	
Co1	0.54906 (8)	0.75790 (4)	0.42739 (5)	0.02739 (19)	
Cl1	1.03803 (16)	0.63906 (7)	0.55162 (10)	0.0487 (4)	
Cl2	0.89886 (13)	0.66652 (6)	0.28277 (8)	0.0347 (3)	
O11	0.6005 (3)	0.67029 (15)	0.4340 (2)	0.0288 (7)	
C11	0.5129 (5)	0.6209 (2)	0.4128 (3)	0.0292 (10)	
C12	0.5785 (5)	0.5615 (2)	0.4072 (3)	0.0304 (11)	
O12	0.7279 (4)	0.56332 (16)	0.4243 (3)	0.0344 (8)	
C121	0.8027 (7)	0.5055 (3)	0.4161 (4)	0.0451 (14)	
H12A	0.7824	0.475	0.4609	0.068*	
H12B	0.9076	0.5136	0.4272	0.068*	
H12C	0.7694	0.4884	0.3547	0.068*	
C13	0.4944 (7)	0.5080 (3)	0.3858 (4)	0.0397 (13)	
H13	0.5397	0.4681	0.3827	0.048*	
C14	0.3425 (7)	0.5123 (3)	0.3688 (4)	0.0441 (15)	
H14	0.2845	0.4756	0.3528	0.053*	
C15	0.2777 (6)	0.5695 (3)	0.3751 (4)	0.0420 (14)	
H15	0.1747	0.5722	0.3644	0.05*	
C16	0.3625 (6)	0.6246 (3)	0.3975 (4)	0.0339 (11)	
C161	0.2874 (5)	0.6845 (3)	0.3990 (4)	0.0388 (13)	
H161	0.1849	0.6823	0.3928	0.047*	
N16	0.3433 (4)	0.7399 (2)	0.4076 (3)	0.0361 (10)	
C162	0.2435 (6)	0.7940 (3)	0.4047 (5)	0.0498 (16)	
H16A	0.1439	0.7784	0.3993	0.075*	
H16B	0.2483	0.8207	0.3521	0.075*	
H16C	0.272	0.8188	0.4607	0.075*	
C21	0.5855 (5)	0.8855 (3)	0.4726 (4)	0.0344 (12)	
O21	0.5157 (4)	0.84486 (17)	0.4129 (3)	0.0351 (8)	
C22	0.6147 (6)	0.9468 (3)	0.4418 (4)	0.0360 (12)	
O22	0.5648 (4)	0.95460 (18)	0.3497 (3)	0.0402 (9)	
C221	0.5993 (7)	1.0125 (3)	0.3126 (4)	0.0464 (14)	
H22A	0.5663	1.0476	0.3457	0.07*	
H22B	0.5507	1.0147	0.2482	0.07*	
H22C	0.7048	1.0154	0.3182	0.07*	
C23	0.6830 (6)	0.9915 (3)	0.5003 (4)	0.0447 (14)	
H23	0.7012	1.032	0.4778	0.054*	
C24	0.7272 (7)	0.9785 (3)	0.5946 (4)	0.0436 (14)	
H24	0.7754	1.0098	0.6354	0.052*	
C25	0.6993 (6)	0.9197 (3)	0.6259 (4)	0.0416 (13)	
H25	0.7275	0.9106	0.6891	0.05*	
C26	0.6302 (6)	0.8730 (3)	0.5665 (4)	0.0358 (12)	
C261	0.5976 (5)	0.8127 (3)	0.6033 (4)	0.0351 (12)	
H261	0.6065	0.8104	0.6673	0.042*	
N26	0.5577 (5)	0.7616 (2)	0.5575 (3)	0.0326 (10)	
C262	0.5263 (7)	0.7067 (3)	0.6084 (4)	0.0427 (13)	
H26A	0.6005	0.6742	0.608	0.064*	
H26B	0.4303	0.6898	0.5801	0.064*	
H26C	0.5269	0.719	0.6713	0.064*	
O31	0.7577 (4)	0.76899 (16)	0.4532 (2)	0.0288 (7)	
C31	0.8156 (5)	0.8150 (2)	0.4098 (4)	0.0296 (11)	
C32	0.9275 (5)	0.8534 (3)	0.4572 (4)	0.0328 (11)	
O32	0.9740 (4)	0.84021 (19)	0.5477 (3)	0.0418 (9)	
C321	1.0785 (7)	0.8820 (3)	0.6003 (4)	0.0517 (16)	
H32A	1.1694	0.88	0.5784	0.078*	
H32B	1.0971	0.8693	0.6645	0.078*	
H32C	1.0408	0.9253	0.5943	0.078*	
C33	0.9856 (6)	0.9006 (3)	0.4114 (4)	0.0404 (13)	
H33	1.0592	0.9275	0.4442	0.048*	
C34	0.9374 (6)	0.9089 (3)	0.3184 (4)	0.0405 (13)	
H34	0.9793	0.9409	0.2877	0.049*	
C35	0.8303 (6)	0.8714 (3)	0.2708 (4)	0.0370 (12)	
H35	0.7988	0.8767	0.2069	0.044*	
C36	0.7665 (6)	0.8248 (2)	0.3164 (4)	0.0317 (11)	
C361	0.6466 (6)	0.7878 (2)	0.2648 (4)	0.0326 (11)	
H361	0.6391	0.7853	0.2008	0.039*	
N36	0.5503 (5)	0.7583 (2)	0.2971 (3)	0.0328 (9)	
C362	0.4337 (6)	0.7279 (3)	0.2312 (4)	0.0405 (13)	
H36A	0.4517	0.7332	0.1697	0.061*	
H36B	0.3403	0.7473	0.2344	0.061*	
H36C	0.431	0.6827	0.2453	0.061*	
O1	0.3489 (6)	0.8820 (3)	0.2162 (4)	0.0631 (13)	
H1AO	0.408 (9)	0.892 (4)	0.265 (4)	0.095*	
H1BO	0.403 (9)	0.878 (5)	0.179 (5)	0.095*	

### Atomic displacement parameters (Å^2^)

**Table:**

	*U*^11^	*U*^22^	*U*^33^	*U*^12^	*U*^13^	*U*^23^
Cd1	0.02341 (18)	0.03144 (19)	0.0287 (2)	0.00034 (13)	0.00698 (13)	−0.00319 (14)
Co1	0.0266 (4)	0.0294 (4)	0.0262 (4)	0.0041 (3)	0.0058 (3)	−0.0003 (3)
Cl1	0.0484 (8)	0.0558 (9)	0.0347 (7)	0.0149 (7)	−0.0076 (6)	−0.0074 (6)
Cl2	0.0320 (6)	0.0434 (7)	0.0309 (6)	0.0019 (5)	0.0116 (5)	−0.0038 (5)
O11	0.0230 (16)	0.0268 (17)	0.038 (2)	−0.0002 (13)	0.0100 (14)	0.0007 (15)
C11	0.032 (3)	0.033 (3)	0.024 (3)	−0.002 (2)	0.009 (2)	0.004 (2)
C12	0.035 (3)	0.033 (3)	0.023 (2)	−0.004 (2)	0.006 (2)	0.001 (2)
O12	0.0344 (19)	0.0258 (18)	0.045 (2)	0.0039 (14)	0.0131 (16)	−0.0004 (16)
C121	0.051 (3)	0.035 (3)	0.052 (4)	0.013 (3)	0.018 (3)	0.005 (3)
C13	0.054 (3)	0.034 (3)	0.032 (3)	−0.006 (2)	0.013 (3)	−0.001 (2)
C14	0.053 (4)	0.048 (3)	0.029 (3)	−0.025 (3)	0.004 (2)	0.001 (2)
C15	0.037 (3)	0.060 (4)	0.030 (3)	−0.016 (3)	0.008 (2)	0.006 (3)
C16	0.030 (3)	0.045 (3)	0.027 (3)	−0.005 (2)	0.008 (2)	0.003 (2)
C161	0.016 (2)	0.065 (4)	0.037 (3)	0.002 (2)	0.008 (2)	0.004 (3)
N16	0.023 (2)	0.045 (3)	0.039 (3)	0.0044 (19)	0.0065 (18)	0.000 (2)
C162	0.029 (3)	0.050 (4)	0.071 (4)	0.013 (3)	0.012 (3)	−0.002 (3)
C21	0.027 (2)	0.039 (3)	0.039 (3)	0.007 (2)	0.012 (2)	−0.006 (2)
O21	0.0331 (19)	0.036 (2)	0.034 (2)	0.0036 (15)	0.0034 (15)	−0.0030 (16)
C22	0.038 (3)	0.031 (3)	0.041 (3)	0.002 (2)	0.013 (2)	−0.003 (2)
O22	0.047 (2)	0.036 (2)	0.038 (2)	0.0058 (17)	0.0084 (17)	0.0001 (17)
C221	0.052 (4)	0.042 (3)	0.048 (4)	0.011 (3)	0.017 (3)	0.007 (3)
C23	0.045 (3)	0.042 (3)	0.049 (4)	−0.003 (3)	0.016 (3)	−0.008 (3)
C24	0.044 (3)	0.045 (3)	0.043 (3)	−0.007 (3)	0.012 (3)	−0.013 (3)
C25	0.035 (3)	0.054 (4)	0.035 (3)	−0.001 (3)	0.007 (2)	−0.008 (3)
C26	0.030 (3)	0.045 (3)	0.034 (3)	0.004 (2)	0.011 (2)	−0.006 (2)
C261	0.030 (3)	0.051 (3)	0.026 (3)	0.004 (2)	0.011 (2)	−0.004 (2)
N26	0.031 (2)	0.042 (3)	0.025 (2)	0.0081 (18)	0.0093 (17)	0.0019 (19)
C262	0.047 (3)	0.047 (3)	0.036 (3)	−0.002 (3)	0.013 (2)	0.004 (3)
O31	0.0298 (17)	0.0287 (17)	0.0291 (18)	0.0003 (14)	0.0084 (14)	0.0012 (14)
C31	0.023 (2)	0.034 (3)	0.034 (3)	0.0031 (19)	0.011 (2)	−0.002 (2)
C32	0.029 (3)	0.034 (3)	0.035 (3)	0.002 (2)	0.008 (2)	0.004 (2)
O32	0.036 (2)	0.051 (2)	0.035 (2)	−0.0085 (17)	0.0009 (16)	0.0033 (18)
C321	0.045 (3)	0.065 (4)	0.043 (4)	−0.016 (3)	0.003 (3)	0.001 (3)
C33	0.033 (3)	0.038 (3)	0.049 (4)	0.000 (2)	0.008 (2)	0.000 (3)
C34	0.036 (3)	0.039 (3)	0.047 (3)	−0.002 (2)	0.008 (2)	0.011 (3)
C35	0.036 (3)	0.038 (3)	0.038 (3)	0.007 (2)	0.010 (2)	0.006 (2)
C36	0.032 (3)	0.032 (3)	0.033 (3)	0.006 (2)	0.011 (2)	0.003 (2)
C361	0.038 (3)	0.030 (3)	0.031 (3)	0.009 (2)	0.008 (2)	−0.001 (2)
N36	0.037 (2)	0.032 (2)	0.028 (2)	0.0012 (18)	0.0016 (18)	−0.0008 (18)
C362	0.046 (3)	0.043 (3)	0.031 (3)	−0.008 (3)	0.004 (2)	−0.004 (2)
O1	0.058 (3)	0.062 (3)	0.063 (3)	0.014 (2)	−0.004 (2)	−0.002 (3)

### Geometric parameters (Å, º)

**Table:**

Cd1—O11	2.235 (3)	C221—H22B	0.98
Cd1—O31	2.286 (3)	C221—H22C	0.98
Cd1—Cl1	2.4091 (14)	C23—C24	1.417 (9)
Cd1—Cl2	2.4222 (12)	C23—H23	0.95
Cd1—O12	2.459 (3)	C24—C25	1.375 (9)
Co1—O21	1.871 (4)	C24—H24	0.95
Co1—O11	1.913 (3)	C25—C26	1.396 (8)
Co1—O31	1.932 (3)	C25—H25	0.95
Co1—N16	1.933 (4)	C26—C261	1.450 (8)
Co1—N26	1.942 (4)	C261—N26	1.293 (7)
Co1—N36	1.961 (5)	C261—H261	0.95
O11—C11	1.327 (6)	N26—C262	1.456 (7)
C11—C16	1.386 (7)	C262—H26A	0.98
C11—C12	1.409 (7)	C262—H26B	0.98
C12—O12	1.374 (6)	C262—H26C	0.98
C12—C13	1.381 (8)	O31—C31	1.349 (6)
O12—C121	1.429 (6)	C31—C36	1.398 (8)
C121—H12A	0.98	C31—C32	1.399 (8)
C121—H12B	0.98	C32—O32	1.367 (7)
C121—H12C	0.98	C32—C33	1.389 (8)
C13—C14	1.400 (9)	O32—C321	1.430 (7)
C13—H13	0.95	C321—H32A	0.98
C14—C15	1.367 (9)	C321—H32B	0.98
C14—H14	0.95	C321—H32C	0.98
C15—C16	1.412 (8)	C33—C34	1.387 (8)
C15—H15	0.95	C33—H33	0.95
C16—C161	1.454 (8)	C34—C35	1.360 (8)
C161—N16	1.279 (8)	C34—H34	0.95
C161—H161	0.95	C35—C36	1.405 (8)
N16—C162	1.474 (7)	C35—H35	0.95
C162—H16A	0.98	C36—C361	1.455 (8)
C162—H16B	0.98	C361—N36	1.277 (7)
C162—H16C	0.98	C361—H361	0.95
C21—O21	1.313 (7)	N36—C362	1.463 (7)
C21—C26	1.410 (8)	C362—H36A	0.98
C21—C22	1.422 (8)	C362—H36B	0.98
C22—C23	1.357 (8)	C362—H36C	0.98
C22—O22	1.374 (7)	O1—H1AO	0.84 (6)
O22—C221	1.413 (7)	O1—H1BO	0.84 (6)
C221—H22A	0.98		
			
O11—Cd1—O31	68.33 (12)	O22—C22—C21	112.9 (5)
O11—Cd1—Cl1	127.90 (10)	C22—O22—C221	116.4 (5)
O31—Cd1—Cl1	111.39 (9)	O22—C221—H22A	109.5
O11—Cd1—Cl2	116.80 (10)	O22—C221—H22B	109.5
O31—Cd1—Cl2	107.72 (9)	H22A—C221—H22B	109.5
Cl1—Cd1—Cl2	112.53 (5)	O22—C221—H22C	109.5
O11—Cd1—O12	66.48 (12)	H22A—C221—H22C	109.5
O31—Cd1—O12	134.52 (12)	H22B—C221—H22C	109.5
Cl1—Cd1—O12	92.53 (9)	C22—C23—C24	120.7 (6)
Cl2—Cd1—O12	96.82 (9)	C22—C23—H23	119.6
O21—Co1—O11	173.23 (16)	C24—C23—H23	119.6
O21—Co1—O31	92.41 (15)	C25—C24—C23	118.8 (5)
O11—Co1—O31	82.67 (14)	C25—C24—H24	120.6
O21—Co1—N16	92.19 (18)	C23—C24—H24	120.6
O11—Co1—N16	92.91 (17)	C24—C25—C26	121.2 (6)
O31—Co1—N16	174.93 (18)	C24—C25—H25	119.4
O21—Co1—N26	92.72 (18)	C26—C25—H25	119.4
O11—Co1—N26	91.78 (17)	C25—C26—C21	120.5 (5)
O31—Co1—N26	88.21 (16)	C25—C26—C261	119.1 (5)
N16—Co1—N26	89.43 (19)	C21—C26—C261	120.3 (5)
O21—Co1—N36	85.17 (17)	N26—C261—C26	126.4 (5)
O11—Co1—N36	90.06 (17)	N26—C261—H261	116.8
O31—Co1—N36	88.82 (17)	C26—C261—H261	116.8
N16—Co1—N36	93.70 (19)	C261—N26—C262	117.1 (5)
N26—Co1—N36	176.3 (2)	C261—N26—Co1	121.0 (4)
C11—O11—Co1	127.9 (3)	C262—N26—Co1	121.8 (4)
C11—O11—Cd1	123.9 (3)	N26—C262—H26A	109.5
Co1—O11—Cd1	104.50 (14)	N26—C262—H26B	109.5
O11—C11—C16	123.7 (5)	H26A—C262—H26B	109.5
O11—C11—C12	117.3 (4)	N26—C262—H26C	109.5
C16—C11—C12	119.0 (5)	H26A—C262—H26C	109.5
O12—C12—C13	125.3 (5)	H26B—C262—H26C	109.5
O12—C12—C11	114.1 (4)	C31—O31—Co1	119.2 (3)
C13—C12—C11	120.6 (5)	C31—O31—Cd1	114.7 (3)
C12—O12—C121	117.6 (4)	Co1—O31—Cd1	102.02 (14)
C12—O12—Cd1	115.7 (3)	O31—C31—C36	120.8 (5)
C121—O12—Cd1	125.1 (3)	O31—C31—C32	120.7 (5)
O12—C121—H12A	109.5	C36—C31—C32	118.6 (5)
O12—C121—H12B	109.5	O32—C32—C33	124.3 (5)
H12A—C121—H12B	109.5	O32—C32—C31	115.8 (5)
O12—C121—H12C	109.5	C33—C32—C31	119.9 (5)
H12A—C121—H12C	109.5	C32—O32—C321	117.5 (5)
H12B—C121—H12C	109.5	O32—C321—H32A	109.5
C12—C13—C14	120.1 (5)	O32—C321—H32B	109.5
C12—C13—H13	120	H32A—C321—H32B	109.5
C14—C13—H13	120	O32—C321—H32C	109.5
C15—C14—C13	119.8 (5)	H32A—C321—H32C	109.5
C15—C14—H14	120.1	H32B—C321—H32C	109.5
C13—C14—H14	120.1	C34—C33—C32	120.8 (5)
C14—C15—C16	120.7 (5)	C34—C33—H33	119.6
C14—C15—H15	119.7	C32—C33—H33	119.6
C16—C15—H15	119.7	C35—C34—C33	120.2 (5)
C11—C16—C15	119.8 (5)	C35—C34—H34	119.9
C11—C16—C161	121.9 (5)	C33—C34—H34	119.9
C15—C16—C161	118.2 (5)	C34—C35—C36	119.9 (5)
N16—C161—C16	127.6 (5)	C34—C35—H35	120
N16—C161—H161	116.2	C36—C35—H35	120
C16—C161—H161	116.2	C31—C36—C35	120.6 (5)
C161—N16—C162	117.6 (5)	C31—C36—C361	120.6 (5)
C161—N16—Co1	124.9 (4)	C35—C36—C361	118.7 (5)
C162—N16—Co1	117.5 (4)	N36—C361—C36	126.3 (5)
N16—C162—H16A	109.5	N36—C361—H361	116.9
N16—C162—H16B	109.5	C36—C361—H361	116.9
H16A—C162—H16B	109.5	C361—N36—C362	116.6 (5)
N16—C162—H16C	109.5	C361—N36—Co1	122.5 (4)
H16A—C162—H16C	109.5	C362—N36—Co1	120.8 (4)
H16B—C162—H16C	109.5	N36—C362—H36A	109.5
O21—C21—C26	124.1 (5)	N36—C362—H36B	109.5
O21—C21—C22	118.6 (5)	H36A—C362—H36B	109.5
C26—C21—C22	117.3 (5)	N36—C362—H36C	109.5
C21—O21—Co1	121.2 (3)	H36A—C362—H36C	109.5
C23—C22—O22	125.6 (5)	H36B—C362—H36C	109.5
C23—C22—C21	121.5 (6)	H1AO—O1—H1BO	103 (9)

### Hydrogen-bond geometry (Å, º)

**Table:**

*D*—H···*A*	*D*—H	H···*A*	*D*···*A*	*D*—H···*A*
C361—H361···Cl1^i^	0.95	2.76	3.509 (5)	137
C362—H36*A*···O32^i^	0.98	2.45	3.204 (7)	134
O1—H1*AO*···O21	0.84 (6)	2.45 (7)	3.140 (6)	139 (9)
O1—H1*AO*···O22	0.84 (6)	2.19 (5)	2.965 (7)	153 (9)
O1—H1*BO*···Cl1^i^	0.84 (6)	2.54 (7)	3.368 (6)	173 (9)

Symmetry code: (i) *x*−1/2, −*y*+3/2, *z*−1/2.
